# Living Root-Mediated Soil Temperature Amplifies the Effects of Experimental Warming on Soil Microarthropod Communities in a *Quercus mongolica* Forest in Northeast China

**DOI:** 10.3390/insects16080809

**Published:** 2025-08-05

**Authors:** Chenglin Chi, Jiannan Wang, Rong Cui, Qianxue Wang, Jili Zhang

**Affiliations:** 1 Harbin Research Institute of Forestry Machinery, National Forestry and Grassland Administration, Harbin 150086, China; chichenglin@caf.ac.cn (C.C.); wangjiannan94@126.com (J.W.); cui126945375@126.com (R.C.); wqx890711@163.com (Q.W.); 2 Research Center of Cold Temperate Forestry, Chinese Academy of Forestry, Harbin 150086, China; 3 National Forestry and Grassland Administration, Heilongjiang Fuyuan Forest Ecosystem Observation and Research Station, Fuyuan 156500, China

**Keywords:** experimental warming, root trenching, temperate forest, soil temperature, soil microarthropod community

## Abstract

The living roots of woody plants in forests play a crucial role in sustaining the soil temperature equilibrium. However, there is limited research investigating the effects of soil temperature balance disruption, influenced by living roots, on soil microarthropods, especially in the context of global climate change. To address this knowledge gap, we conducted a three-year in situ simulation experiment involving either experimental warming or root trenching treatments to mimic environmental changes and their impacts on soil microarthropod communities in a temperate forest ecosystem in Northeast China. Statistical analysis focused on assessing the abundance and family richness of Collembola and Acari. Warming increased soil temperature, while root trenching had contrasting effects. In the absence of root trenching, warming positively influenced Collembola but negatively affected Acari. Conversely, when combined with root trenching, warming had a diminished impact on both Collembola and Acari. Our findings demonstrate that the interactive effects of warming on soil microarthropod communities vary depending on the presence or absence of root trenching. Specifically, within the context of root trenching treatment compared to no-root trenching treatment, warming exhibited a comparatively attenuated influence on soil microarthropod communities. Overall, living roots play a pivotal role in mediating soil temperature conditions, which significantly impact soil microarthropod communities in the context of global climate change.

## Simple Summary

Forest root systems are vital for maintaining soil temperature balance. However, the understanding of how climate change-induced alterations in temperature balance, mediated by living root interactions, affect small soil organisms such as mites and springtails, remains limited. Scientists examined the effects of experimental warming and root trenching, as well as their interaction, on soil microarthropod communities by simulating these conditions in a temperate forest of Northeast China. The findings indicate that warming alone significantly increases the abundance of springtails while simultaneously reducing the abundance of mites. In contrast, root trenching alleviates the negative effects of warming on these soil organisms. This study underscores the critical role of living roots in modulating the attenuation of soil organisms’ responses to climate-induced temperature variations. These results further highlight the urgent need to address the detrimental impacts of root–climate interactions on soil biodiversity, ensuring its preservation in the face of global climate change.

## 1. Introduction

As the foundation of terrestrial ecosystem functioning, soil biodiversity plays a fundamental role in ecosystem processes, functionality and the provision of ecosystem services [1]. Within soil ecosystems, the super-diverse community of microarthropods plays a pivotal role in performing essential functions such as decomposition and nutrient mineralization [2,3,4]. Collembola and mites are representative soil microarthropods that play a crucial role in regulating the community structure of soil microorganisms through direct predation [5,6]. Additionally, these organisms contribute to the fragmentation of litter, thereby increasing the surface area available for microbial colonization [7,8]. This enhanced contact between microorganisms and organic matter indirectly accelerates litter decomposition and nutrient cycling [7,8]. Numerous collembolan species demonstrate superior adaptation to high moisture environments because of their specialized physiological structures, such as the tracheal system and continuous lipid/wax cecal layer [9]; however, they are more susceptible to drought and temperature fluctuations than other microarthropods [10,11]. The diverse responses exhibited by these groups may lead to alterations in community composition and soil fauna diversity, thereby influencing their ecosystem functions [12,13,14]. Consequently, soil microarthropod communities are widely regarded as robust model organisms for quantifying soil biodiversity [15,16]. Among these organisms, soil microarthropods constitute a particularly sensitive bioindicator group due to their narrow ecological niches. For an extended period, researchers have investigated the responses of soil microarthropod communities to local and regional disturbances, such as logging [17]. However, owing to their pivotal role in delivering ecosystem services, interest in the study of their response to global climate change (such as warming) is progressively growing [18].

The global surface temperature increased by 0.84–1.10 °C from 2001 to 2020, compared to the reference period of 1850–1900 [19]. Climate warming is expected to accelerate decomposition and nutrient cycling rates, thereby enhancing subterranean biomass through increased metabolic activity of soil microorganisms [20,21]. This process is further mediated by warming-induced shifts in living roots; warming may initially stimulate root exudation (e.g., sugars, organic acids) and fine root turnover, providing labile carbon to fuel microbial proliferation [22,23]. Changes in soil microbial communities induced by warming are anticipated to indirectly affect soil fauna communities via trophic cascades [24]. Furthermore, climate change is projected to directly impact the metabolic processes [25], development [26], survival and reproduction of soil fauna [27], as well as alter interactions among these communities. These interconnected shifts in community interactions could cascade through trophic networks, jeopardizing the stability of soil ecosystem services critical to forest health. Therefore, gaining a more comprehensive understanding of the responses of soil microarthropods to climate change will facilitate the accurate prediction of the future state of forest ecosystems.

Soil moisture and temperature are intricately interconnected in regulating subsurface processes, with living roots of forest plants serving as a critical medium that facilitates this coupling [28]. Living roots actively mediate water transport between soil layers via hydraulic redistribution (HR), maintain rhizosphere hydration and directly influence soil thermal properties [29]. Moist soil exhibits high heat capacity and thermal inertia, effectively dampening temperature fluctuations [30]. Conversely, water uptake by living roots reduces soil moisture content. This reduction in soil moisture, coupled with diminished evaporative cooling capacity [31], increases ecosystem vulnerability to extreme heat events exacerbated by warming [32]. These bidirectional interactions result in the formation of distinct rhizosphere microclimates, where temperature and moisture are dynamically regulated by biological activities [33]. For example, root exudates not only promote soil aggregation and modify pore structure but also enhance water retention while reducing thermal conductivity [34]. Additionally, infiltration near the root zone creates large pores that facilitate vertical water movement, establishing local moisture gradients and enabling heat dissipation through potential transfer mechanisms [35]. This synergistic effect allows living roots to function as ecological engineers by reconfiguring the temperature–moisture relationship [36], ultimately determining the suitability of microhabitats for soil microarthropods.

Root trenching is widely utilized in soil ecology research, encompassing investigations into nutrient transformation [37,38,39], soil chemical properties [21,40,41], organic matter decomposition [42], soil respiration [43] and above-ground productivity [44,45]. Yet, the potential of this method to elucidate root-mediated soil temperature effects on microarthropod communities—a critical knowledge gap in climate change ecology—has rarely been explored.

This knowledge gap is particularly significant because living roots may act as a “Janus-faced element” in modulating the effects of warming. The active root system serves as an ecological buffer by stabilizing microhabitat structures to alleviate thermal stress [46], but it may also exert the opposite effect under certain conditions. In the context of warming, this root-mediated temperature regulation mechanism is anticipated to play an increasingly critical role by continuously disrupting and reconfiguring the coupling between soil temperature and moisture dynamics, as rising temperatures intensify soil desiccation risks and modify hydrological cycles [47]. Since soil microarthropods are highly dependent on rhizosphere-mediated microhabitat conditions (e.g., moisture gradients and thermal buffering), understanding how these root-driven processes interact with warming to shape faunal communities is critical. However, our current understanding of how living roots interact with warming to influence soil fauna remains limited and fragmented, especially regarding the specific responses of distinct microarthropod groups.

To address this question, we conducted an operational experiment spanning three years to scrutinize the response of soil microarthropod model groups (Collembola and Acari) regarding their mobile activity under the influence of warming and root trenching. Temperate forests in northeast China are located within the high-latitude climate zone of the Northern Hemisphere, exhibiting heightened vulnerability to climate change [48]. Temperature is the primary limiting factor in this region [49,50]. We hypothesized that (i) Collembola would display a more pronounced response to warming than Acari, (ii) Collembola responded more strongly to root trenching than Acari and (iii) root trenching suppressed the effects of warming on soil microarthropod communities.

## 2. Materials and Methods

### 2.1. Site Description

The study area was located in the hinterland of the Sanjiang Plain (47°25′ N, 133°40′ E) within the eastern region of Heilongjiang Province, China. It encompasses a low-mountainous and hilly topography, with elevations ranging from 50 to 278 m. The area exhibits a temperate continental monsoon climate, characterized by cold and arid winters and hot and humid summers. The average annual precipitation is 600 mm, and the average yearly temperature stands at 2.2 °C, with a frost-free period lasting approximately 150 days. The plant composition in this region is predominantly East Siberian flora, which consists mainly of temperate broad-leaved forests exhibiting zonal vegetation patterns. The dominant tree includes *Quercus mongolica*, *Populus davidiana*, *Betula platyphylla*, *Fraxinus mandshurica*, *Phellodendron amurense* and *Juglans mandshurica*. Understory shrubs are primarily *Lespedeza bicolor*, *Corylus heterophylla*, *Spiraea salicifolia* and *Syringa oblata*. The prevailing soil types in this region include dark brown, loamy and marshy soils [51].

### 2.2. Experimental Design and Set-Up

**Figure A1:**
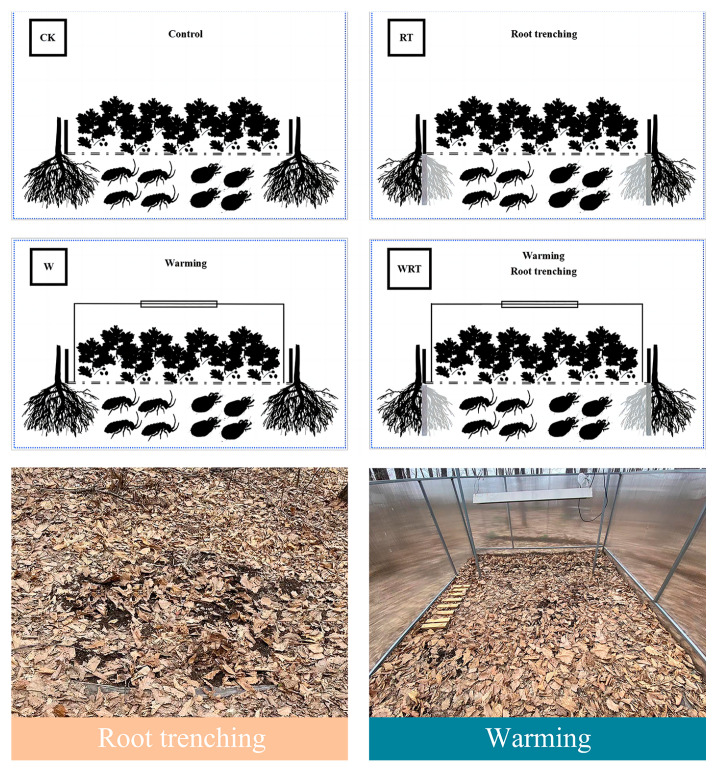
The experimental design of warming and root trenching. Control (CK), warming (W), root trenching (RT) and combined treatment (WRT) were established within a *Quercus mongolica* forest.

The experimental site was established in a natural Mongolian oak forest in 2020, and the treatments were implemented after the system had stabilized. Intensive monitoring was conducted from July to September 2023. This experiment comprised four treatments: control (CK), warming (W), root trenching (RT) and warming plus root trenching (WRT). A completely randomized block design with three replicates was employed, resulting in a total of 12 sample plots, each measuring 2 m × 3 m. The three replicates represent spatially independent plots, and all within-plot measurements were aggregated to the plot level before statistical analysis was conducted. Each plot was separated from adjacent plots by a minimum distance of 150 m. For the root trenching treatment, to exclude disturbances from roots outside the plots, the perimeters were trenched to a depth of 100 cm and lined with thick plastic sheets. To simulate warming, open-top chambers (OTCs) equipped with infrared radiators (Kalglo Electronics, Bethlehem, PA, USA) were used as warming devices. The OTCs were situated 15 cm above the soil surface. The triangular prism-shaped lamp body measured 165 cm in length and 15 cm in width, while the cylindrical lamp tube had dimensions of 150 cm in length and 8 mm in diameter. The reflector surface of the radiator was suspended at a height of 150 cm above the warming plots. To minimize the influx of live root carbon, herbs in both treated and control plots were pruned five times annually (detailed experimental design information is listed in Figure A1) [52].

### 2.3. Soil Microclimate Measurements

An automatic soil temperature and moisture recorder (Em50 Digital Data Logger, Decagon Devices Inc., Pullman, WA, USA) continuously monitored soil temperature and volumetric water content at a depth of 0–10 cm on a monthly basis from July to September 2023, with measurements taken three times daily (at 8 a.m., noon and 4 p.m.) for three consecutive days. Monthly mean values for each treatment were obtained by averaging the daily means of soil temperature and volumetric water content.

### 2.4. Sampling, Extraction and Identification of Soil Microarthropods

Soil fauna samples were collected once a month from July to September 2023, encompassing three distinct time periods. This intervention mitigated the resource scarcity resulting from the initial trench effect [53] and facilitated the relative stability of soil fauna [54]. Within each plot, three sampling points were randomly taken at each sampling occasion. The soil collection procedure involved using a 4 cm diameter and 14 cm height soil auger, allowing for sample collection within the 0–10 cm depth range. Subsequently, the meticulously gathered samples were packed into plastic bags for transportation to the laboratory.

The three soil cores from each plot were combined to create a composite sample before microarthropod extraction. Subsequently, 375 mL of the soil sample was placed onto a 20-mesh screen and subjected to Tullgren extraction. Soil microarthropods were extracted over a period of 168 h using Tullgren funnels equipped with a 25 W light source, with specimens directed downward into collection bottles containing 75% ethanol. Post-extraction inspection of soil cores was conducted to verify complete specimen recovery. Microarthropod classification to the family level was achieved by using a microscope (Nikon 80i, Minato City, Japan) at approximately 200× magnification [55,56,57,58,59].

### 2.5. Statistical Analysis

First, we performed data visualization to examine the changes in the soil microclimate and spatial distribution of soil microarthropods. Additionally, unsupervised pattern recognition principal component analysis (PCA) was employed to explore the differences and variability among all soil microarthropods, providing an initial understanding. The composition of soil microarthropod communities was determined using sample-based substitution multiple variance analysis (PERMANOVA) with a Bray–Curtis distance matrix (using the ‘vegan’ package version 2.5-6 in R).

The generalized linear mixed model (GLMM) was employed to assess the effects of warming and root trenching on soil temperature, soil volumetric water content, taxa abundance and family richness of microarthropods (Collembola, Oribatida and Mesostigmata). Warming, root trenching and their interaction were included as fixed effects, while sampling time was treated as a random effect. Poisson distribution, with logarithmic link functions, was employed to analyze family richness data. In contrast, a Gaussian distribution, with identity link functions, was utilized to explore soil temperature, soil volumetric water content data and taxa abundance data. Post hoc LSD tests were conducted to identify significant differences among the respective levels within the factors.

The soil microarthropod discrimination among treatments was accomplished using Partial Least Squares Discriminant Analysis (PLS-DA) with supervised pattern recognition. Taxa abundance of soil microarthropods, identified through VIP values greater than 1, Student’s *t*-test with a significance level of *p* < 0.05 and Log_2_FC values equal to or exceeding two or less than or equal to 0.5 were employed for family selection.

Since the gradient length calculated by the first DCA axis (2.1 SD) fell below the threshold of 3.0 SD, we employed a constrained linear model (RDA) to explore the association between environmental factors and variations in soil microarthropod communities. Furthermore, we conducted 999 Monte Carlo permutation tests to concurrently evaluate the significance of these differences.

Partial Least Squares Path Modeling (PLS-PM) was employed to investigate and evaluate the direct and indirect impacts of warming, root trenching and related soil temperature and volumetric water content on the abundance of taxa and family richness of Collembola and Acari. Goodness of Fit (GOF) values and *R*^2^ coefficients were utilized as assessment indicators, with *R*^2^ values representing the proportion of each variable’s explained variance and GOF reflecting how well the entire model fits the data.

To minimize the potential bias introduced by rare species in the results, the weights of certain rare species (e.g., Acaridae sp1) were adjusted downward during the analysis. All data analyses and visualizations were performed using R version 3.6.3.

## 3. Results

### 3.1. Variation in Soil Microclimate

**Figure 1:**
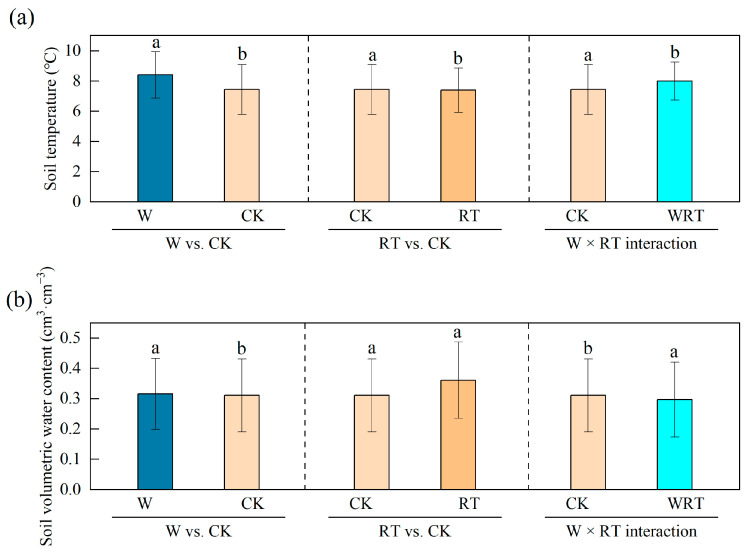
Variation in the soil microclimate (EMM ± SE) in WRT, W, RT and CK. (**a**) Soil temperature and (**b**) soil volumetric water content. WRT: warming plus root trenching; W: warming; RT: root trenching; CK: control. Different lowercase letters within the group denote statistically significant differences among treatments.

As anticipated, the warming treatment yielded a substantial elevation in soil temperature by 1.02 °C (Table A1, Figure 1a). Furthermore, the warming treatment significantly decreased soil volumetric water content (Figure 1b, Table 1), regardless of whether root trenching was applied. On the other hand, the implementation of root trenching had a significant impact on reducing soil temperature (Figure 1a, Table 1). Still, it did not show any significant effect on soil volumetric water content (Figure 1b, Table 1).

### 3.2. Taxa Abundance and Family Richness of Soil Microarthropods Under Warming and Root Trenching Treatments

In this study, we collected 637 individuals from four families of Collembola and 1045 individuals from seven families of Acari (Table A2). Among the Acari, the identified specimens belonged to three suborders (Oribatida-non Astigmatina, Mesostigmata and Oribatida-Astigmatina; Table A2). For simplicity, these mite groups are subsequently referred to as Oribatida, Mesostigmata and Astigmatina, respectively. The dominant families in Collembola were Onychiuridae (25.6%), Neanuridae (23.9%) and Isotomidae (43.2%). The dominant families in Acari were Digamasellidae (36.1%), Oppiidae (16.2%) and Nothridae (21.0%).

**Figure 2:**
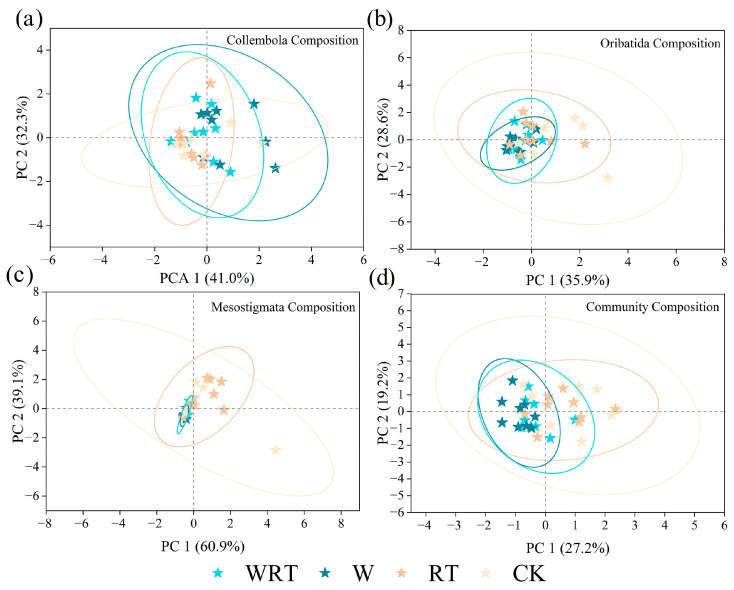
Principal component analysis (PCA) plots of the Acari and Collembola families. (**a**) Collembola, (**b**) oribatid mites, (**c**) mesostigmatid mites and (**d**) the entire soil microarthropod community in WRT, W, RT and CK. WRT: warming plus root trenching; W: warming; RT: root trenching; CK: control. Stars of the same color belong to the same group, as indicated by an oval; each star corresponds to a specific sample (**a**–**d**).

Principal component analysis (PCA) results indicated that neither warming nor root trenching significantly impacted the composition of the collembolan community (Figure 2a; Adonis, *p* = 0.469). However, the combined treatment of warming and root trenching significantly affected the composition of both the oribatid (Figure 2b; Adonis, *p* = 0.034) and mesostigmatid communities (Figure 2c; Adonis, *p* = 0.034). Furthermore, these treatments significantly influenced the overall composition of the soil microarthropod community (Figure 2d; Adonis, *p* = 0.001).

**Figure 3:**
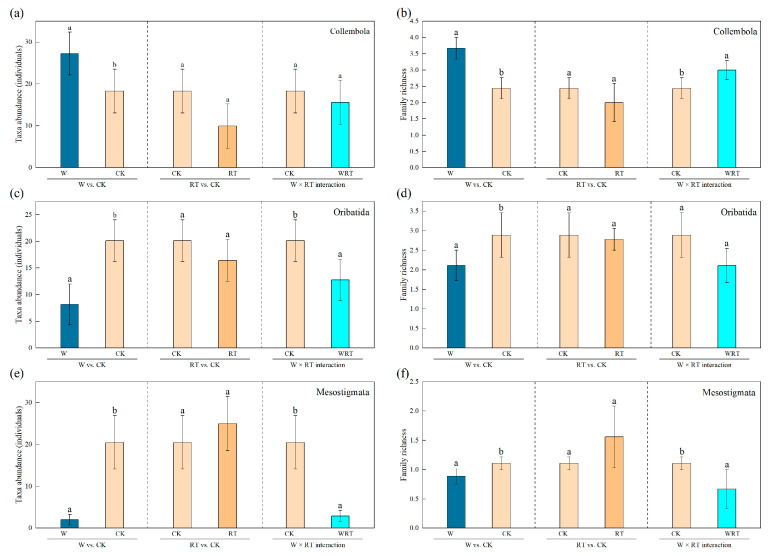
Effect of warming (W), root trenching (RT) and their interactions (W × RT) on taxa abundance and family richness (EMM ± SE) of soil microarthropods (Collembola, Oribatida and Mesostigmata). WRT: warming plus root trenching; W: warming; RT: root trenching; CK: control. Different lowercase letters within the group denote statistically significant differences among treatments. Panels (**a**,**b**), (**c**,**d**) and (**e**,**f**), respectively depict taxa abundance and family richness in Collembola, Oribatida and Mesostigmata.

The warming treatment significantly increased taxa abundance (30.9%) and family richness of Collembola (Figure 3a,b; Table 2). Additionally, the warming treatment also resulted in a significant decrease in the abundance and family richness of Oribatida and Mesostigmata, respectively (36.6% and 78.7%; Figure 3c–f; Table 2). However, root trenching did not significantly impact the taxa abundance and family richness of Collembola, Oribatida and Mesostigmata (Figure 3a–f; Table 2). Warming plus root trenching treatments significantly enhanced family richness and marginally increased the taxa abundance of Collembola (6.5%; Figure 3a,b; Table 2). Conversely, warming plus root trenching significantly reduced the taxa abundance (21.0% and 60.7%) and family richness of Oribatida and Mesostigmata, respectively (Figure 3c–f; Table 2).

### 3.3. Predominant Expression of Family of Soil Microarthropods Under Different Treatments

**Figure 4:**
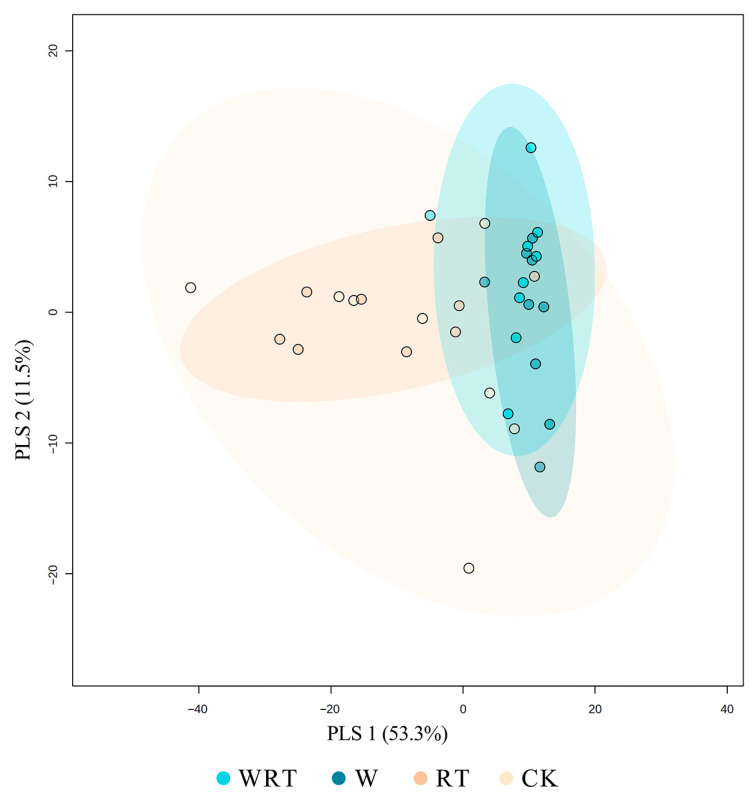
Partial Least Squares Discriminant Analysis (PLS-DA) of soil microarthropod communities in different treatments. WRT: warming plus root trenching; W: warming; RT: root trenching; CK: control. The groups of samples subjected to different treatments are represented by circles of varying colors or shapes. The horizontal and vertical axes were scaled regarding relative distance and practical significance.

Partial Least Squares Discriminant Analysis (PLS-DA) revealed significant disparities in the community structure of soil microarthropods across different treatments (Figure 4). PLS 1 (53.3%) was identified as the primary factor responsible for segregating the WRT, W, RT and CK groups (Figure 4; *R*^2^X = 0.407, *R*^2^Y = 0.241 and *Q*^2^ = 0.0175). The minimal distinctions observed among the CK, RT, W and WRT groups resulted in an overlap between the four treatments. Through comparative analysis, Digamasellidae (VIP=1.68), Neanuridae (VIP = 1.22) and Oppiidae (VIP = 1.02) were identified as key families exhibiting differential expression across various treatments (Figure 4).

### 3.4. The Relationship Between Soil Microclimate and Soil Microarthropod Community

**Figure 5:**
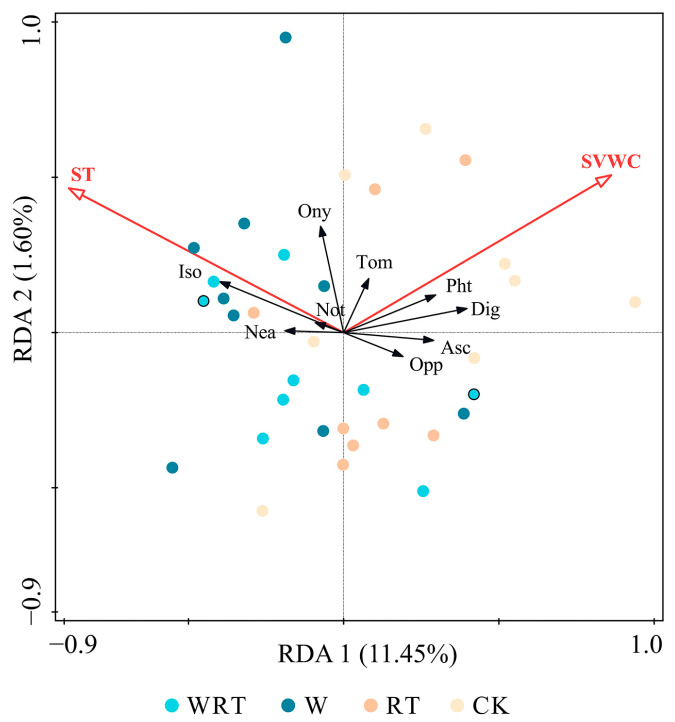
Redundancy analysis (RDA) suggesting associations between soil microarthropod communities and soil microclimate variables. WRT, warming plus root trenching; W, warming; RT, root trenching; CK, control; ST, soil temperature; SVWC, soil volumetric water content; Asc, Ascidae; Dig, Digamasellidae; Iso, Isotomidae; Nea, Neanuridae; Not, Nothruidae; Opp, Oppiidae; Ony, Onychiuridae; Pht, Phthiracaridae; Tom, Tomoceridae.

The effect of soil microclimate factors on soil microarthropod communities was assessed using redundancy analysis (Figure 5). The first axis demonstrates that soil temperature significantly influences 11.5% and 1.6% of the variations in the community composition of soil microarthropods. The composition of the soil microarthropod family varied in response to different warming and root trenching treatments, suggesting that these organisms have significant potential as indicators of temperature fluctuations at the family level (Figure 5). The observed increase in spatial separation between distinct treatments provided compelling evidence of significant divergence in soil microclimate conditions (Figure 5). In the RDA plot depicting the soil microclimate (Figure 5), Neanuridae, Isotomidae, Nothruidae and Onychiuridae were positively correlated with soil temperature, whereas Phthiracaridae, Digamasellidae, Ascidae and Oppiidae were negatively correlated with it. These findings suggest that soil temperature is the primary determinant influencing the distribution patterns of Neanuridae, Isotomidae, Nothruidae, Onychiuridae, Phthiracaridae, Digamasellidae, Ascidae and Oppiidae; however, Tomoceridae demonstrated statistically non-significant constraints within the RDA model (Figure 5).

### 3.5. Contributions of Warming, Root Trenching and Soil Temperature to Soil Microarthropod Community

**Figure 6:**
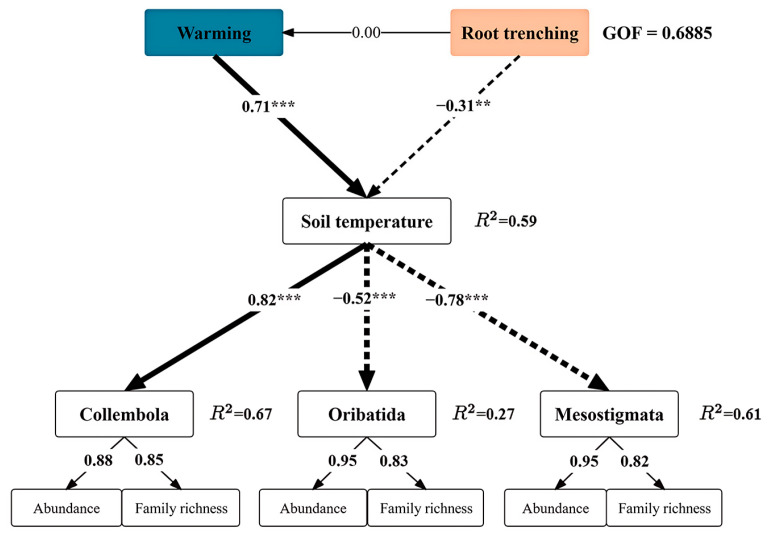
Partial Least Squares Path Modeling (PLSPM) was employed to investigate the relationships between warming, root trenching and soil temperature with taxa abundance and family richness of microarthropods (Collembola, Oribatida and Mesostigmata). The solid line represents a positive relationship, while the dashed line indicates a negative relationship. The varying thickness of the lines correspondingly signify the strength and significance of the path effect. Standardized coefficients are indicated by numbers beside arrows. Model evaluation was conducted using goodness of fit (GOF), while *R*^2^ values represent the proportion of explained variance for each variable. Levels of significance were denoted as ** for *p* < 0.01 and *** for *p* < 0.001.

The initial development of a PLS-PM model (GOF = 0.6885) aimed to establish connections among warming, root trenching, soil temperature, Collembola, Oribatida and Mesostigmata (Figure 6). The findings revealed significant direct or indirect relationships between warming, root trenching and soil temperature. The model effectively captured a substantial portion (59.0%) of the observed variability in soil temperature while also providing comprehensive explanations for variations in population sizes among Collembola (67.0%), Oribatida (27.0%) and Mesostigmata (61.0%). The warming treatment exhibited a significantly positive influence on soil temperature (*p* < 0.001), whereas the root trenching treatment demonstrated a significantly negative impact on soil temperature (*p* < 0.01). Soil temperature showed a significant positive correlation with collembolan communities (*p* < 0.001) while displaying significant negative correlations with oribatid and mesostigmatid communities (*p* < 0.001). Warming and root trenching indirectly influenced the diversity of collembolan, oribatid and mesostigmatid communities through their direct impact on soil temperature.

## 4. Discussion

By investigating the impacts of experimental warming and root trenching on soil microarthropod communities in a temperate natural Mongolian oak forest in China, we have observed that the warming treatment directly influences both beneficial and detrimental aspects of these communities. However, root trenching does not significantly affect soil microarthropod communities.

### 4.1. Effects of Experimental Warming

Our study demonstrates that the collembolan community exhibited a significant positive response to warming, indicating that short-term temperature increases may have beneficial effects on collembolan species. With the intensification of evaporation from soil surfaces, elevated temperatures often induce aridity, leading to a shift in the community composition of Collembola towards species that prefer low moisture [60]. However, redundancy analysis (RDA) results revealed that warming did not exert any discernible influence on the water preference of the collembolan community. This lack of response may be attributed to the fact that even under the most extreme warming scenarios, soil moisture levels remain relatively high in a temperate forest of China [49,50]. Furthermore, studies indicate that the warming phenomenon may counterbalance the adverse impacts of drought on Neanuridae species, thereby enhancing their survival prospects [61]. Therefore, we conclude that moisture conditions may not have limited the survival of Collembola in this habitat. The observed increases in taxa abundance and family richness of Collembola can be partially attributed to their smaller body size. The universal metabolic scaling rule grants small species a thermal advantage at higher temperatures due to their accelerated developmental rates and superior maximum population growth rates compared to larger species [62]. These findings align with prior studies indicating that elevated temperatures may positively impact collembolan populations residing in temperate forests [63].

Unlike the effects on Collembola, our findings demonstrate a significant reduction in the taxa abundance of Acari due to warming. One plausible explanation is that these organisms actively avoid high temperatures to mitigate heat stress [64,65,66]. The decline in cold-adapted species as a result of warming has led to substantial changes in the composition of soil microarthropod communities [64]. Additionally, there are cascading effects that depend on external water sources beyond the root zone, such as the observed decline in soil nematode populations, which serve as a critical food source for other organisms [67].

Apparently, Collembola stands to gain greater advantages from warming compared to Acari, thereby supporting our first hypothesis. In general, the influence of warming and associated growth strategies may induce alterations in soil microarthropod communities due to changes in their resilience.

### 4.2. Effects of Root Trenching

We observed that root trenching had no significant effect on the abundance of most soil microarthropod species and there were no notable changes in the community composition of soil microarthropods. This finding challenges our second hypothesis that Acari would be less responsive to root trenching than Collembola. Our results corroborate prior findings [39], indicating that soil water availability exerts a similar degree of influence on both springtails and mites. A plausible explanation for this phenomenon is that environmental filtering may select assemblages with greater resilience to fluctuating moisture conditions [68].

In contrast to the impact on soil microarthropods, our results from generalized linear mixed models indicate a positive yet non-significant influence of root trenching on soil moisture levels. The observed increase in soil water content may be attributed to root grooves, which impede water absorption by roots and consequently lead to an accumulation of soil water within these grooves. This finding aligns with previous studies demonstrating the beneficial effect of root trenching on soil quality in forest ecosystems [38,41,69,70]. However, it is worth noting that our results also reveal a detrimental impact of root trenching on soil temperature. The observed reduction in soil temperature within the root gully can primarily be attributed to the endothermic effects of soil water evaporation [71]. Additionally, the concurrent decline in biomass and reduced activity of soil microbiota [39,72] outside the experimental context has led to decreased heat generation from microbial decomposition processes [73].

Interestingly, in our study, the microclimate (soil temperature and soil moisture) directly influenced by the root trench does not appear to impact Collembola or Acarid populations. A likely explanation for this phenomenon is that Collembola and Acarid species may have evolved buffering mechanisms, such as epidermal water retention capabilities [9], to adapt to short-term microclimate fluctuations. Consequently, the temperature and moisture changes induced by root trenching have not exceeded their adaptive thresholds.

### 4.3. Effects of Experimental Warming and Root Trenching

The presence of root trenching, as revealed by structural equation modeling (SEM), caused a decrease in soil temperature. This reduction subsequently suppressed springtail abundance but promoted mite abundance. This is evidenced by the less pronounced increase in temperature at the warming treatment site with root trenching compared to the control site without root trenching, as well as the relatively minor impact of warming on both the taxa abundance and family richness of soil microarthropods. This finding aligns with our third hypothesis. One plausible explanation is that plant root metabolic products, such as root exudates, root-induced soil modifications and hydraulic redistribution, interact with soil structure to enhance soil water retention and maintain moisture equilibrium [74]. The rapid endothermic evaporation of soil water, coupled with decreased soil temperature due to reduced root metabolic activities [75], mitigates the positive effects of warming on soil microarthropods.

In summary, soil temperature emerges as the primary determinant elucidating alterations in the abundance of specimens and richness of families of soil microarthropods. The observed interactions between root trenching and warming treatments on soil microarthropod abundance and community composition suggest that the positive effects of a single factor on soil microarthropods may diminish when another factor is concurrently changing. This counteraction may be attributed to the excessive reduction in soil temperature under the dual-factor treatment. The root trenching treatment alleviated the thermal stress on soil microarthropod communities, which suppressed the collembolan community and enhanced the mite community.

This study investigated the effects of warming and living root interactions on soil microclimate variability and the community structure of soil microarthropods, offering insights into how these factors jointly influence belowground ecosystems. However, several limitations exist, including the relatively short experimental duration, the restriction of the research scope to a single climate zone and the insufficient exploration of high-resolution quantification of microclimate dynamics. These aspects collectively constrain the depth of our understanding regarding how microclimate thresholds shape species distribution patterns. Future research should integrate high-frequency soil monitoring technologies with cross-regional comparative analyses conducted across diverse climatic zones, thereby facilitating a comprehensive assessment of the adaptability of the soil hydrological–biological network in response to climate change.

### 4.4. Study Limitations and Future Research

It is important to note that although the number of experimental replicates in this study was sufficient to detect the main and interactive treatment effects reported, it remained relatively limited due to the logistical complexity of the in situ manipulations and the intensive sampling regime. This limitation may affect the precision of effect size estimation and restrict the broader applicability of our findings to the full spatial variability within *Quercus mongolica* forests or across temperate forest ecosystems in general.

Future research should prioritize replicating these manipulations across a broader spatial scale and increasing plot-level replication to more effectively capture ecosystem-level variability and improve the generalizability of the observed patterns. Long-term monitoring across diverse forest types would further clarify the broader ecological implications under projected climate warming scenarios.

## 5. Conclusions

When considering the interaction between experimental warming and root trenching, it is evident that the effect of root trenching buffers the impact of warming on soil microarthropod communities in temperature-limited forests. Specifically, the implementation of root trenching significantly attenuated the positive impact of warming on the collembolan community and alleviated the negative impact on the mite community. Our data suggest a relatively muted response of soil microarthropods to the inhibition of living root activity while underscoring the indispensable role of living roots in forest ecosystems. Specifically, living roots play a pivotal role in mediating soil moisture conditions, which have a substantial impact on soil microarthropod communities in the context of global climate change.

## Figures and Tables

**Table 1:** *F* and *p*-values from Generalized Linear Mixed Model (GLMM) of the effect of warming (W), root trenching (RT) and their interactions on soil temperature and volumetric water content. Bold numbers indicate significant differences (*p* < 0.05).

Variable	Soil Temperature			Soil Volumetric Water Content	
	df	*F* Value	*p*	*F* Value	*p*
W	1, 33	48.679	**0.000**	11.616	**0.002**
RT	1, 33	10.882	**0.002**	0.008	0.930
W × RT	3, 32	19.502	**0.000**	4.855	**0.007**

**Table 2:** *F* and *p*-values from Generalized Linear Mixed Model (GLMM) of the effect of warming (W), root trenching (RT) and their interactions on taxa abundance and family richness (log_10_(N + 1)) of soil microarthropods (Collembola, Oribatida and Mesostigmata). Bold numbers indicate significant differences (*p* < 0.05).

Variable	Collembola					Oribatida				Mesostigmata			
	Taxa Abundance			Family Richness		Taxa Abundance		Family Richness		Taxa Abundance		Family Richness	
	df	*F* Value	*p*	*F* Value	*p*	*F* Value	*p*	*F* Value	*p*	*F* Value	*p*	*F* Value	*p*
W	1, 33	5.606	**0.024**	14.233	**0.001**	7.559	**0.010**	4.638	**0.039**	51.950	**0.000**	11.861	**0.002**
RT	1, 33	2.899	0.098	2.844	0.101	0.233	0.632	0.024	0.878	1.945	0.172	2.274	0.141
W × RT	3, 32	2.833	0.054	5.521	**0.004**	3.194	**0.037**	1.554	0.220	17.334	**0.000**	6.639	**0.001**

## Data Availability

The data presented in this study are available on request from the corresponding author. The data are not publicly available due to privacy restrictions.

## Appendix A

**Table A1:** Soil temperature and soil volumetric water content. WRT: warming plus root trenching; W: warming; RT: root trenching; CK: control. The values are the estimated marginal means (EMM) ± standard error (SE).

Parameters	CK	W	RT	WRT
Soil temperature (°C)	7.4 ± 1.6	8.4 ± 1.5	7.3 ± 1.5	8.0 ± 1.3
Soil volumetric water content (cm^3^∙cm^−3^)	0.3 ± 0.1	0.3 ± 0.1	0.4 ± 0.1	0.3 ± 0.1

**Table A2:** Groups and quantities of soil microarthropods. WRT: warming plus root trenching; W: warming; RT: root trenching; CK: control. The values are the estimated marginal means (EMM) ± standard error (SE).

Groups		CK	W	RT	WRT
Collembola	Onychiuridae	7.4 ± 8.2	5.9 ± 3.0	1.4 ± 2.1	3.3 ± 2.2
	Neanuridae	3.2 ± 5.1	6.7 ± 3.9	2.4 ± 4.9	4.6 ± 4.8
	Isotomidae	5.7 ± 7.4	12.9 ± 11.3	5.1 ± 5.5	6.9 ± 7.5
	Tomoceridae	1.7 ± 3.0	1.8 ± 1.8	1.0 ± 1.3	0.8 ± 0.7
Oribatida	Phthiracaridae	4.2 ± 5.3	1.3 ± 1.9	2.1 ± 3.1	1.9 ± 1.8
	Oppiidae	5.4 ± 4.6	1.7 ± 1.7	6.1 ± 3.2	5.6 ± 7.2
	Nothridae	7.2 ± 6.3	4.9 ± 3.1	6.9 ± 5.8	5.3 ± 6.0
	Protoribatidae	3.2 ± 2.4	0.3 ± 1.0	1.3 ± 1.5	0.0 ± 0.0
Mesostigmata	Digamasellidae	16.4 ± 17.1	1.9 ± 2.5	20.8 ± 12.7	2.8 ± 4.8
	Ascidae	4.0 ± 10.9	0.1 ± 0.3	4.2 ± 4.0	0.1 ± 0.3
Astigmata	Acaridae	3.6 ± 5.4	1.9 ± 2.9	2.1 ± 4.9	0.7 ± 1.0
